# Supplementary material for: Different phylotypes of Cutibacterium acnes cause different modic changes in intervertebral disc degeneration
Source: PLoS One. 2022 Jul 12;17(7):e0270982. doi: 10.1371/journal.pone.0270982 (PMC9275720; doi:10.1371/journal.pone.0270982)
Supplement: Attachment — Submitted filename: Response to reviewers.docx [file pone.0270982.s002.docx]

Dear editor and reviewers,

Thank you for your letter and the reviwers’ comments concerning our manuscript. We are happy to improve the manuscript based on these helpful and valuable comments from you. We have revised the manuscript according to the editor’s advice, hoping to meet the submission requirements. Revisions in the text are shown using red highlight for additions, and strikethrough font for deletions. The responses to the reviewer's comments are presented following.

We would love to thank you for allowing us to resubmit a revised copy of the manuscript and we highly appreciate your time and consideration.

Sincerely,

Haichuan Lu.

Reviewer #1: I disagree that different biomechanical environment of the animal model, justifies proposing primates as the next step. MMP part should be strengthen if it is to be included and reported as results.

Response: Thank you for your constructive suggestion. Indeed primates model might not be strictly required in this research, we will seriously consider about the animals in future correlational study. We add more interpretation regarding MMP in the text.

Reviewer #1: P Acnes name has been changed to Cutibacterium acnes, I’d suggest you to at least mention that change either on introduction and/or discussion.

Response: We agree with the suggestion and have changed “*P. acnes”* to “*C. acnes*” throughout the text. Thank you.

Reviewer #1: Animal groups nomination is confusing in the manuscript, sometimes using Groups I, II, III and other times you refer to the bacteria type. Revise.

Response: We apologize for the confusion generated by the previous manuscript and we have corrected the group nomination in related description of three groups of animals, using group I, II, III instead of bacteria type in the text. In the statement of culture experiment, we use the bacteria type rather than the groups.

Reviewer #1: How did you measure discal MR signal intensity? Describe your method and the software utilized.

Response: We are grateful for the advice. We have added more details of MR signal intensity in methods.

Reviewer #1: Page 7, initial paragraph, I guess it should say “Since the avascular…”, not vascular.

Response: We have corrected the word in our manuscript. Thank you.

Reviewer #1: The role of MMP in the pathogenesis of the model is not well introduced, It’s methods for detection are not described and yet, is one of the main results. Please, amend. What’s the hypothetical contribution of MMP to the pathogenesis of MCs?

Response: We deeply appreciate the reviewer’s suggestion. We have added more interpretation regarding MMP in the part of introduction and methods of the article.

MMPs were increased in nucleus pulposus cells under disc degeneration, the biological characteristics of early cartilaginous endplate degeneration are extracellular matrix degradation and release of MMPs. It has been suggested that *C. acnes* infection inhibits lubricin expression by cartilage endplate cells and the expression of MMP-1 is increased, thus *C. acnes* may facilitate the progression of MCs [Liu J, Wei X, Huang B, Wu H, Zhang X, Chen J,et al. Lubricin expression in the lumbar endplate and its association with Modic changes. J Orthop Translat. 2019 Nov 15;22:124-131]. And MMP-13 were increased when cartilaginous endplate cells was stimulated with *C. acnes* supernatant [Zhang Y, Wang Y, Yuan Y, Lin Y, Lin B, Zhou H. Propionibacterium acnes induces cartilaginous endplate degeneration by promoting MIF expression via the NF-κB pathway. J Orthop Surg Res. 2020 Jun 9;15(1):213].

Reviewer #1: Why did you use type IB P Acnes instead of IA1? (actually, you had more cases of IA1). Please, clarify.

Response: We are so grateful for the suggestion. Originally, we decided to use type IB *P Acnes* just because strain type IA has been studied by other investigators and they found increased degeneration by inoculation of caudal discs with strain IA of *P Acnes* [Zamora T, Palma J, Andia M, Garcia P, Wozniak A, Solar A, Campos M. Effect of Propionibacterium acnes (PA) injection on intervertebral disc degeneration in a rat model: Does it mimic modic changes? Orthop Traumatol Surg Res. 2017 Sep;103(5):795-799]. Though phylotype IB may be less prevalent than type IA, we supposed this strain of *P Acnes* also needs to be studied. Even so, we indeed should add this type of *P Acnes* in our research. All of strains we identified would be included in our future investigation.

Reviewer #1: On the part of the discussion, I would moderate the conclusion about type 1-2 P. Acnes producing Modic 1 changes and type 3, Modic 3, since this study has limited numbers of experimental animals for each type. And outside this study, there is no evidence to support such affirmation. I would rephrase this part more on the side of data suggesting that different strains of P Acnes are capable of producing discal damage differently.

Response: We are very grateful to reviewer for pointing out this problem. We have rephrased this part as following:“The results of our study indicated that infection of different phylotypes of C. acnes might be capable of producing discal damage differently. We speculated one of the possible reasons may be the different virulence of various species of C. acnes, some of them are more virulent, but others may be less virulent [26], thus probably leading to different pathological change and dispersion of MRI image. Therefore, we supported that the intervertebral discs infected by C. acnes would have more chance to result in latent low-grade chronic inflammation[27], which is then represented as Modic changes and disc degeneration rather than the pyogenic discitis.”

Reviewer #1: A section of study limitations is missing: low number of subjects (for a prevalence study), not having negative control cultures (i.e. from surgical field), low number of animals per group and thus, using solely the results of the current study, one cannot rule out that P Acnes might be a contaminant. As noted on the discussion as reasons, none of them are contained in this paper. Please add a limitation section on your discussion.

Response:Thank you for your careful review. A limitation section has been added in discussion as following: “In addition, the sample sizes of both clinical research and animal experiment were relatively small. We agree that even after stringent preoperative skin disinfection, contamination of biopsy samples with skin microbiota cannot be fully excluded. In the part of clinical sample collection, some muscle and ligaments around the disc should be obtained as control tissue in consideration of rigorousness. Further comprehensive investigations about this issue both in vivo and in vitro should be conducted in the future.”

Reviewer #2: In this report, authors isolate C.acnes from human discs after surgical excision and culturing with a 30% positivity rate. Apparently there was no control tissue in this point of the research to determine rate of positive disc colonization by C. acnes, and not contamination during the process. Otherwise, there is controversy as well in controlling culture with surrounding disc tissues (muscles, ligament) since the defect of the annulus could be a way to bacterial spread of the C. acnes outside the disc. Although this is a weakness to determine rate of true disc colonization, the isolated phylotypes of C. acnes were inoculated into rats intervertebral disc and it does not seem to invalidate this process.

Response: We are very grateful to your comments. Contamination is the biggest reason to question the presence of C. acnes in nonpyogenic intervertebral discs. Some tissue samples adjacent to the intervertebral discs indeed could serve as markers of contamination during surgery. We should consider this research more comprehensively. The limitation has been added in the discussion part of the paper. As the reviewer says, we could still believe *C. acnes* has a strong connection with disc degeneration considering the following inoculated diseased experimental host. Thank you again for the constructive advice.

Reviewer #2:Methods says that patients' pain were accessed but there is no report in the results section about it. If this is not an analyzed measure, it should not be in methods. How long were patients followed after primary surgery?

Response: Thank you for underlining this deficiency. The statement about subjects has been improved in Methods of the article. We conducted 1-year follow-up study of the patients and the clinical parameters would be in another prospective study concerning C. ances and post-surgical patients.

Reviewer #2: Percutaneous puncture: it is not clear if after percutaneous puncture of the disc, the rats were opened with a surgical approach to the vertebra and then bone wax was placed at the punctures’ holes. It says that the rat was sutured layer by layer giving the impression that a surgical approach was performed.

Response: We apologize for the puzzled description of puncture, indeed there were no surgical approach after percutaneous puncture. It was confused with another animal study and we have revised it in the method part of manuscript. Thanks so much for your suggestion.

Reviewer #2: It does not prove or gives an answer to the whole C. acnes presence in the disc or its relationship to disc degeneration, but it is original in stating the effects of the C. acnes and some of their different phylotypes in normal disc of rats.

Response: Thank you so much for your careful summary. We deeply appreciate the reviewer’s positive evaluation of our work. There are many limitations in the research and our findings about the different phylotypes of C.acnes still remains to be elucidated. Though there are some shortage of funding, we will make more efforts to improve the study in the future investigation. Thank you again for your precious comments and advice.
